# 14-(1,3-Benzodioxol-5-yl)-7,14-dihydro­dibenzo[*a*,*j*]acridine

**DOI:** 10.1107/S1600536810044302

**Published:** 2010-11-06

**Authors:** Runhong Jia, Juhua Peng, ShuJiang Tu

**Affiliations:** a Lianyungang Teachers’ College, Lianyungang 222006, People’s Republic of China; b College of Chemistry and Chemical Engineering, Xuzhou Normal University, Xuzhou 221116, People’s Republic of China

## Abstract

The title compound, C_28_H_19_NO_2_, was synthesized by the reaction of 1,3-benzodioxole-5-carbaldehyde with naphthalen-2-amine catalyzed by thio­salicylic acid in acetic acid. The central dihydropyridine ring adopts a boat conformation. The two planar (r.m.s. deviations = 0.0158 and 0.0552 Å) bicyclic parts make a dihedral angle of 16.16 (5)° with respect to each other. The crystal packing is stabilized by inter­molecular N—H⋯O hydrogen bonds and C—H⋯π inter­actions.

## Related literature

For a similar crystal structure, see: Ray *et al.* (1995 ▶). For the applications of charge-transport materials, see: Marder *et al.* (2005 ▶). For the use of dihydro­acridine derivatives as therapeutic agents, see: Rudler *et al.* (2008 ▶). For their biological activities, see Ellis & Stevens (2001 ▶). For literature on this class of compound, see: Llama *et al.* (1989 ▶). For literature on drug development, see: Khurana *et al.* (1990 ▶). For puckering parameters, see: Cremer & Pople (1975 ▶).
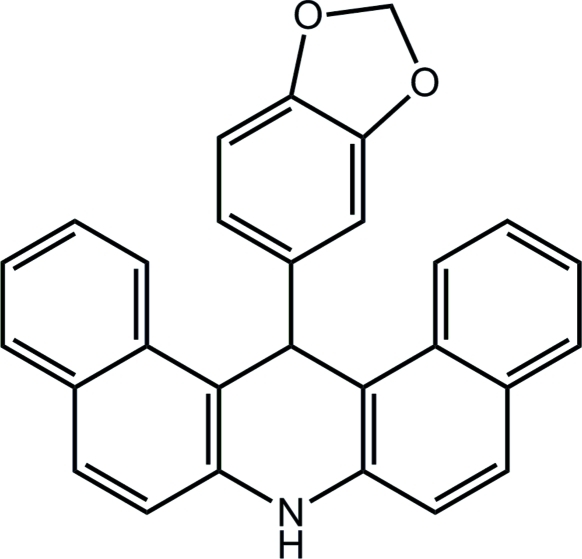


## Experimental

### 

#### Crystal data

C_28_H_19_NO_2_
*M* *_r_* = 401.44
Monoclinic, 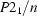

*a* = 9.4920 (11) Å
*b* = 11.2767 (16) Å
*c* = 18.883 (2) Å
β = 102.650 (2)°
*V* = 1972.1 (4) Å^3^
*Z* = 4
Mo *K*α radiation
μ = 0.09 mm^−1^
*T* = 298 K
0.18 × 0.12 × 0.10 mm

#### Data collection

Bruker SMART CCD area-detector diffractometer
Absorption correction: multi-scan (SABABS; Sheldrick, 1996 ▶) *T*_min_ = 0.985, *T*_max_ = 0.992
10175 measured reflections
3484 independent reflections
1877 reflections with *I* > 2σ(*I*)
*R*_int_ = 0.059

#### Refinement

*R*[*F* ^2^ > 2σ(*F* ^2^)] = 0.042
*wR*(*F* ^2^) = 0.054
*S* = 1.03
3484 reflections
280 parameters
H-atom parameters constrained
Δρ_max_ = 0.15 e Å^−3^
Δρ_min_ = −0.15 e Å^−3^

### 

Data collection: *SMART* (Bruker, 1998 ▶); cell refinement: *SAINT* (Bruker, 1999 ▶); data reduction: *SAINT*; program(s) used to solve structure: *SHELXS97* (Sheldrick, 2008 ▶); program(s) used to refine structure: *SHELXL97* (Sheldrick, 2008 ▶); molecular graphics: *SHELXTL* (Sheldrick, 2008 ▶); software used to prepare material for publication: *SHELXTL*.

## Supplementary Material

Crystal structure: contains datablocks global, I. DOI: 10.1107/S1600536810044302/zq2067sup1.cif

Structure factors: contains datablocks I. DOI: 10.1107/S1600536810044302/zq2067Isup2.hkl

Additional supplementary materials:  crystallographic information; 3D view; checkCIF report

## Figures and Tables

**Table 1:** Hydrogen-bond geometry (Å, °). *Cg* is the centroid of the C13–C18 ring.

*D*—H⋯*A*	*D*—H	H⋯*A*	*D*⋯*A*	*D*—H⋯*A*
N1—H1⋯O2^i^	0.86	2.31	3.108 (2)	154
C5—H5⋯*Cg*^ii^	0.93	2.90	3.793 (2)	161

Symmetry codes: (i) 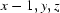; (ii) 
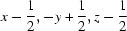
.

**Fig. 1.**
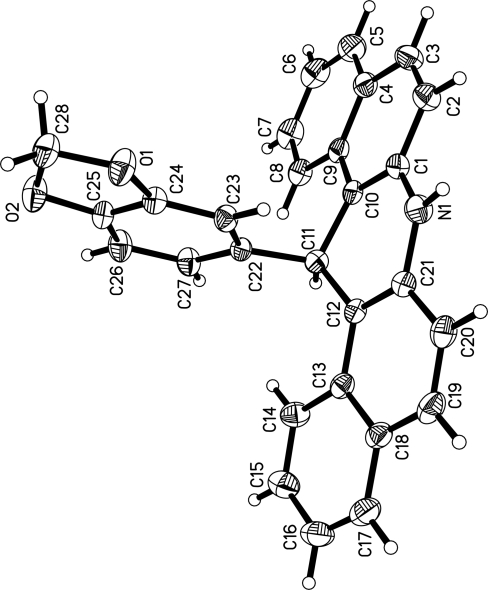
The molecular structure of the title compound, showing 30% probability displacement ellipsoids.

**Fig. 2.**
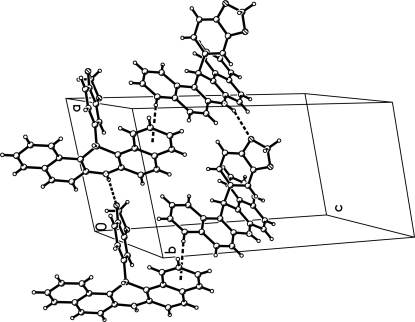
The packing diagram of the title compound viewed along the a axis.

## supplementary crystallographic information

### Comment

The charge-transport materials can be used in organic electronic devices such as organic light-emitting diodes, lasers, photovoltaic cells, photodetectors, active and passive electronic devices, and memories (Marder *et al.*, 2005). The extended angular fused aza-heterocycles (V-type fused aza-heterocycles) exhibit important photophysics properties, which are widely applied in the charge-transport materials due to their strong skeleton rigidity and large conjugation systems. With large conjugation systems, dibenzacridine derivatives, especially the acridinium ions, possess interesting photophysical properties such as the presence of intramolecular electron-transfer state of a high energy and long lifetime, which have been tested and applied as an efficient photocatalyst in modeling the photosynthetic reactions. Furthermore, dihydroacridine derivatives with an 1,4-DHPs parent nucleus are well known as therapeutic agents (Rudler *et al.*, 2008). Due to their interesting biological activities such as antimalarial and antitumor, they have immense utility in pharmaceutical industry (Ellis *et al.*, 2001). Therefore, this class of compounds has been the focus of much recent research (Llama *et al.*, 1989), and has led to intensive interest in the synthesis of several drugs based on them (Khurana *et al.*, 1990). For these reasons, the synthesis of dihydroacridine with an 1,4-DHPs parent nucleus is strongly desired.

In the title molecule (Fig. 1), the dihydropyrimidine ring system is in a boat conformation. The puckering parameters (Cremer & Pople, 1975) are q_2_ = 0.240 (2) Å, and φ_2_ = 174.3 (5)°, Q = 0.248 (2) Å and θ = 75.7 (5) °. Besides, the distances between atoms N1 and C11, and the mean plane C1/C10/C12/C21 (r.m.s. deviation = 0.012 Å) are 0.133 (2) and 0.286 Å, which also confirm the conformation of the pyridine ring. The dihedral angle between the aforementioned weighted plane and phenyl ring of C22—C27 is 85.66 (7)°, which shows that the two units are nearly perpendicular. The two planar bicyclic parts make a dihedral angle of 16.16 (5) with respect to each other, which is smaller than that of the previously reported crystal structure of 14-methyl-7,14-dihydrodibenzo[a,j]acridine (Ray *et al.*, 1995).

The crystal packing is stabilized by intermolecular N—H···O hydrogen bonds and C—H···π interactions (Table 1, Fig.2).

### Experimental

The title compound was prepared by the reaction of 1,3-benzodioxole-5-carbaldehyde (1 mmol) and naphthalen-2-amine (2 mmol), with thiosalicylic acid (1 mmol) as catalyst in acetic acid (1.5 ml). Single crystals were obtained by slow evaporation of a 95% aqueous ethanol solution (yield 75%; m.p. >573 K). IR (cm^-1^): 3406.0, 3020.6, 1587.9, 1530.5, 1484.6, 1246.2, 1033.7, 922.0, 807.1, 746.4. ^1^H NMR (DMSO-d_6_): 9.54 (s, 1H, NH), 8.56 (d, J = 8.4 Hz, 2H, ArH), 7.79 (d, J = 8.0 Hz, 2H, ArH), 7.75 (d, J = 8.8 Hz, 2H, ArH), 7.52 (t, J = 7.6 Hz, 2H, ArH), 7.35 (d, J = 8.4 Hz, 2H, ArH), 7.29 (t, J = 7.2 Hz, 2H, ArH), 7.12–7.08 (m, 2H, ArH), 6.64 (d, J = 7.6 Hz, 2H, ArH), 6.63 (s, 1H, CH), 5.77 (s, 2H, CH_2_)

### Refinement

All H atoms were positioned geometrically and treated as riding, with N—H = 0.86 Å and C—H = 0.93–0.97 Å, and included in the final cycles of refinement using a riding model, with *U*_iso_(H) = 1.2*U*_eq_(parent atom).

### Crystal data

**Table:**

C_28_H_19_NO_2_	*D*_x_ = 1.352 Mg m^−^^3^
*M**_r_* = 401.44	Melting point = 522–524 K
Monoclinic, *P*2_1_/*n*	Mo *K*α radiation, λ = 0.71073 Å
*a* = 9.4920 (11) Å	Cell parameters from 1646 reflections
*b* = 11.2767 (16) Å	θ = 2.7–25.3°
*c* = 18.883 (2) Å	µ = 0.09 mm^−^^1^
β = 102.650 (2)°	*T* = 298 K
*V* = 1972.1 (4) Å^3^	Block, yellow
*Z* = 4	0.18 × 0.12 × 0.10 mm
*F*(000) = 840	

### Data collection

**Table:**

Bruker SMART CCD area-detector diffractometer	3484 independent reflections
Radiation source: fine-focus sealed tube	1877 reflections with *I* > 2σ(*I*)
graphite	*R*_int_ = 0.059
φ and ω scans	θ_max_ = 25.0°, θ_min_ = 2.1°
Absorption correction: multi-scan (SABABS; Sheldrick, 1996)	*h* = −10→11
*T*_min_ = 0.985, *T*_max_ = 0.992	*k* = −9→13
10175 measured reflections	*l* = −22→20

### Refinement

**Table:**

Refinement on *F*^2^	Primary atom site location: structure-invariant direct methods
Least-squares matrix: full	Secondary atom site location: difference Fourier map
*R*[*F*^2^ > 2σ(*F*^2^)] = 0.042	Hydrogen site location: inferred from neighbouring sites
*wR*(*F*^2^) = 0.054	H-atom parameters constrained
*S* = 1.03	*w* = 1/[σ^2^(*F*_o_^2^) + (0.003*P*)^2^] where *P* = (*F*_o_^2^ + 2*F*_c_^2^)/3
3484 reflections	(Δ/σ)_max_ < 0.001
280 parameters	Δρ_max_ = 0.15 e Å^−^^3^
0 restraints	Δρ_min_ = −0.14 e Å^−^^3^

### Special details

**Table:**

Geometry. All e.s.d.'s (except the e.s.d. in the dihedral angle between two l.s. planes) are estimated using the full covariance matrix. The cell e.s.d.'s are taken into account individually in the estimation of e.s.d.'s in distances, angles and torsion angles; correlations between e.s.d.'s in cell parameters are only used when they are defined by crystal symmetry. An approximate (isotropic) treatment of cell e.s.d.'s is used for estimating e.s.d.'s involving l.s. planes.
Refinement. Refinement of *F*^2^ against ALL reflections. The weighted *R*-factor *wR* and goodness of fit *S* are based on *F*^2^, conventional *R*-factors *R* are based on *F*, with *F* set to zero for negative *F*^2^. The threshold expression of *F*^2^ > σ(*F*^2^) is used only for calculating *R*-factors(gt) *etc*. and is not relevant to the choice of reflections for refinement. *R*-factors based on *F*^2^ are statistically about twice as large as those based on *F*, and *R*- factors based on ALL data will be even larger.

### Fractional atomic coordinates and isotropic or equivalent isotropic displacement parameters (Å^2^)

**Table:**

	*x*	*y*	*z*	*U*_iso_*/*U*_eq_	
N1	0.45989 (16)	0.20369 (16)	0.04518 (9)	0.0462 (5)	
H1	0.4105	0.1523	0.0631	0.055*	
O1	0.99695 (14)	0.01008 (13)	0.12539 (7)	0.0528 (4)	
O2	1.20015 (14)	0.07828 (14)	0.08905 (8)	0.0558 (5)	
C1	0.4820 (2)	0.18618 (19)	−0.02422 (11)	0.0381 (6)	
C2	0.3990 (2)	0.0986 (2)	−0.06736 (12)	0.0497 (6)	
H2	0.3326	0.0545	−0.0487	0.060*	
C3	0.4150 (2)	0.0779 (2)	−0.13573 (13)	0.0532 (7)	
H3	0.3610	0.0186	−0.1634	0.064*	
C4	0.5134 (2)	0.1458 (2)	−0.16540 (12)	0.0447 (6)	
C5	0.5262 (2)	0.1275 (2)	−0.23780 (12)	0.0589 (7)	
H5	0.4712	0.0690	−0.2658	0.071*	
C6	0.6184 (2)	0.1946 (2)	−0.26691 (12)	0.0623 (8)	
H6	0.6264	0.1817	−0.3145	0.075*	
C7	0.7013 (2)	0.2831 (2)	−0.22529 (12)	0.0576 (7)	
H7	0.7642	0.3285	−0.2456	0.069*	
C8	0.6910 (2)	0.30386 (19)	−0.15506 (11)	0.0458 (6)	
H8	0.7465	0.3633	−0.1284	0.055*	
C9	0.5960 (2)	0.23510 (19)	−0.12254 (11)	0.0380 (6)	
C10	0.58159 (19)	0.25377 (18)	−0.04931 (11)	0.0335 (5)	
C11	0.68212 (19)	0.33592 (18)	0.00266 (10)	0.0345 (5)	
H11	0.6959	0.4083	−0.0238	0.041*	
C12	0.61922 (19)	0.37069 (19)	0.06732 (10)	0.0331 (5)	
C13	0.6730 (2)	0.46985 (19)	0.11175 (11)	0.0374 (6)	
C14	0.7842 (2)	0.5444 (2)	0.09827 (11)	0.0474 (6)	
H14	0.8244	0.5294	0.0585	0.057*	
C15	0.8335 (2)	0.6380 (2)	0.14276 (12)	0.0580 (7)	
H15	0.9064	0.6859	0.1326	0.070*	
C16	0.7766 (3)	0.6629 (2)	0.20290 (12)	0.0602 (7)	
H16	0.8115	0.7268	0.2328	0.072*	
C17	0.6702 (2)	0.5938 (2)	0.21792 (12)	0.0550 (7)	
H17	0.6319	0.6111	0.2580	0.066*	
C18	0.6164 (2)	0.4957 (2)	0.17369 (11)	0.0420 (6)	
C19	0.5078 (2)	0.4219 (2)	0.19024 (11)	0.0506 (7)	
H19	0.4698	0.4385	0.2305	0.061*	
C20	0.4587 (2)	0.3275 (2)	0.14816 (11)	0.0479 (7)	
H20	0.3875	0.2794	0.1598	0.057*	
C21	0.5148 (2)	0.3012 (2)	0.08644 (11)	0.0378 (6)	
C22	0.82907 (19)	0.27519 (19)	0.02735 (10)	0.0333 (5)	
C23	0.8363 (2)	0.17159 (19)	0.06878 (10)	0.0374 (6)	
H23	0.7561	0.1442	0.0847	0.045*	
C24	0.9643 (2)	0.11199 (19)	0.08518 (10)	0.0356 (6)	
C25	1.0846 (2)	0.1529 (2)	0.06323 (11)	0.0385 (6)	
C26	1.0822 (2)	0.2545 (2)	0.02452 (11)	0.0484 (6)	
H26	1.1644	0.2825	0.0107	0.058*	
C27	0.9509 (2)	0.31549 (19)	0.00623 (10)	0.0422 (6)	
H27	0.9456	0.3849	−0.0208	0.051*	
C28	1.1380 (2)	−0.0228 (2)	0.11716 (11)	0.0524 (7)	
H28A	1.1318	−0.0892	0.0839	0.063*	
H28B	1.1974	−0.0461	0.1636	0.063*	

### Atomic displacement parameters (Å^2^)

**Table:**

	*U*^11^	*U*^22^	*U*^33^	*U*^12^	*U*^13^	*U*^23^
N1	0.0464 (12)	0.0452 (14)	0.0529 (13)	−0.0096 (10)	0.0238 (10)	0.0007 (10)
O1	0.0437 (10)	0.0507 (12)	0.0685 (11)	0.0108 (8)	0.0221 (8)	0.0212 (9)
O2	0.0379 (9)	0.0599 (13)	0.0721 (11)	0.0095 (9)	0.0178 (8)	0.0188 (9)
C1	0.0336 (13)	0.0388 (16)	0.0412 (15)	0.0018 (12)	0.0068 (12)	−0.0004 (12)
C2	0.0463 (15)	0.0457 (18)	0.0574 (17)	−0.0052 (13)	0.0118 (13)	−0.0005 (14)
C3	0.0491 (15)	0.0473 (18)	0.0576 (17)	−0.0048 (13)	−0.0004 (13)	−0.0091 (14)
C4	0.0413 (14)	0.0477 (18)	0.0416 (15)	0.0067 (13)	0.0015 (12)	−0.0028 (13)
C5	0.0602 (17)	0.064 (2)	0.0474 (17)	0.0077 (15)	0.0002 (13)	−0.0054 (14)
C6	0.074 (2)	0.076 (2)	0.0361 (15)	0.0201 (17)	0.0112 (14)	0.0007 (15)
C7	0.0629 (17)	0.071 (2)	0.0428 (16)	0.0125 (15)	0.0199 (14)	0.0116 (14)
C8	0.0458 (14)	0.0505 (17)	0.0427 (15)	0.0053 (12)	0.0132 (12)	0.0051 (12)
C9	0.0332 (13)	0.0407 (16)	0.0395 (14)	0.0097 (12)	0.0069 (11)	0.0027 (12)
C10	0.0283 (12)	0.0343 (15)	0.0375 (14)	0.0064 (11)	0.0063 (11)	0.0024 (11)
C11	0.0368 (13)	0.0321 (15)	0.0360 (13)	−0.0006 (11)	0.0111 (10)	0.0026 (10)
C12	0.0317 (13)	0.0317 (15)	0.0377 (13)	0.0037 (11)	0.0115 (11)	0.0035 (11)
C13	0.0385 (14)	0.0357 (16)	0.0381 (14)	0.0078 (12)	0.0085 (11)	0.0034 (11)
C14	0.0573 (16)	0.0391 (17)	0.0471 (15)	−0.0036 (13)	0.0142 (13)	−0.0011 (12)
C15	0.0632 (17)	0.0503 (19)	0.0568 (17)	−0.0076 (14)	0.0049 (14)	−0.0035 (14)
C16	0.0711 (19)	0.047 (2)	0.0538 (18)	0.0056 (15)	−0.0063 (15)	−0.0119 (14)
C17	0.0616 (17)	0.057 (2)	0.0444 (16)	0.0192 (15)	0.0065 (14)	−0.0062 (14)
C18	0.0445 (15)	0.0429 (17)	0.0373 (14)	0.0107 (13)	0.0062 (12)	−0.0017 (12)
C19	0.0493 (15)	0.062 (2)	0.0454 (16)	0.0121 (14)	0.0213 (13)	−0.0013 (13)
C20	0.0419 (14)	0.0581 (19)	0.0500 (16)	−0.0008 (13)	0.0237 (12)	0.0014 (13)
C21	0.0354 (13)	0.0391 (16)	0.0399 (14)	0.0010 (12)	0.0102 (11)	−0.0017 (12)
C22	0.0303 (12)	0.0365 (15)	0.0350 (13)	−0.0004 (11)	0.0110 (11)	−0.0012 (11)
C23	0.0326 (13)	0.0418 (16)	0.0410 (14)	−0.0025 (12)	0.0152 (11)	0.0018 (11)
C24	0.0356 (13)	0.0369 (16)	0.0355 (13)	0.0006 (12)	0.0104 (11)	0.0067 (11)
C25	0.0312 (13)	0.0431 (17)	0.0413 (14)	0.0067 (12)	0.0080 (11)	0.0047 (12)
C26	0.0354 (13)	0.0579 (19)	0.0559 (16)	−0.0014 (13)	0.0182 (12)	0.0131 (13)
C27	0.0367 (13)	0.0427 (16)	0.0484 (14)	−0.0020 (12)	0.0120 (12)	0.0118 (12)
C28	0.0466 (15)	0.0515 (19)	0.0596 (17)	0.0128 (13)	0.0128 (13)	0.0121 (13)

### Geometric parameters (Å, °)

**Table:**

N1—C21	1.382 (2)	C12—C21	1.372 (2)
N1—C1	1.386 (2)	C12—C13	1.424 (3)
N1—H1	0.8600	C13—C14	1.416 (2)
O1—C24	1.375 (2)	C13—C18	1.420 (2)
O1—C28	1.4297 (19)	C14—C15	1.367 (3)
O2—C25	1.384 (2)	C14—H14	0.9300
O2—C28	1.437 (2)	C15—C16	1.389 (3)
C1—C10	1.377 (2)	C15—H15	0.9300
C1—C2	1.407 (3)	C16—C17	1.354 (3)
C2—C3	1.353 (2)	C16—H16	0.9300
C2—H2	0.9300	C17—C18	1.412 (3)
C3—C4	1.415 (3)	C17—H17	0.9300
C3—H3	0.9300	C18—C19	1.412 (3)
C4—C5	1.414 (3)	C19—C20	1.349 (3)
C4—C9	1.416 (3)	C19—H19	0.9300
C5—C6	1.361 (3)	C20—C21	1.415 (2)
C5—H5	0.9300	C20—H20	0.9300
C6—C7	1.401 (3)	C22—C27	1.380 (2)
C6—H6	0.9300	C22—C23	1.399 (2)
C7—C8	1.371 (2)	C23—C24	1.363 (2)
C7—H7	0.9300	C23—H23	0.9300
C8—C9	1.426 (2)	C24—C25	1.377 (2)
C8—H8	0.9300	C25—C26	1.357 (3)
C9—C10	1.435 (2)	C26—C27	1.399 (2)
C10—C11	1.524 (2)	C26—H26	0.9300
C11—C12	1.523 (2)	C27—H27	0.9300
C11—C22	1.532 (2)	C28—H28A	0.9700
C11—H11	0.9800	C28—H28B	0.9700
			
C21—N1—C1	121.99 (18)	C15—C14—H14	119.4
C21—N1—H1	119.0	C13—C14—H14	119.4
C1—N1—H1	119.0	C14—C15—C16	121.1 (2)
C24—O1—C28	105.07 (15)	C14—C15—H15	119.5
C25—O2—C28	104.73 (15)	C16—C15—H15	119.5
C10—C1—N1	120.3 (2)	C17—C16—C15	119.8 (2)
C10—C1—C2	122.0 (2)	C17—C16—H16	120.1
N1—C1—C2	117.8 (2)	C15—C16—H16	120.1
C3—C2—C1	120.5 (2)	C16—C17—C18	121.1 (2)
C3—C2—H2	119.7	C16—C17—H17	119.5
C1—C2—H2	119.7	C18—C17—H17	119.5
C2—C3—C4	120.4 (2)	C17—C18—C19	121.2 (2)
C2—C3—H3	119.8	C17—C18—C13	119.7 (2)
C4—C3—H3	119.8	C19—C18—C13	119.1 (2)
C5—C4—C3	120.5 (2)	C20—C19—C18	120.7 (2)
C5—C4—C9	120.2 (2)	C20—C19—H19	119.7
C3—C4—C9	119.3 (2)	C18—C19—H19	119.7
C6—C5—C4	120.5 (2)	C19—C20—C21	120.4 (2)
C6—C5—H5	119.7	C19—C20—H20	119.8
C4—C5—H5	119.7	C21—C20—H20	119.8
C5—C6—C7	120.1 (2)	C12—C21—N1	120.74 (19)
C5—C6—H6	119.9	C12—C21—C20	121.4 (2)
C7—C6—H6	119.9	N1—C21—C20	117.9 (2)
C8—C7—C6	120.9 (2)	C27—C22—C23	119.66 (18)
C8—C7—H7	119.5	C27—C22—C11	121.97 (18)
C6—C7—H7	119.5	C23—C22—C11	118.25 (17)
C7—C8—C9	120.6 (2)	C24—C23—C22	118.09 (18)
C7—C8—H8	119.7	C24—C23—H23	121.0
C9—C8—H8	119.7	C22—C23—H23	121.0
C4—C9—C8	117.6 (2)	C23—C24—O1	128.30 (19)
C4—C9—C10	119.8 (2)	C23—C24—C25	121.5 (2)
C8—C9—C10	122.5 (2)	O1—C24—C25	110.14 (18)
C1—C10—C9	117.90 (19)	C26—C25—C24	121.9 (2)
C1—C10—C11	119.70 (19)	C26—C25—O2	128.45 (19)
C9—C10—C11	122.12 (18)	C24—C25—O2	109.64 (18)
C12—C11—C10	111.88 (16)	C25—C26—C27	117.18 (18)
C12—C11—C22	111.21 (15)	C25—C26—H26	121.4
C10—C11—C22	108.92 (16)	C27—C26—H26	121.4
C12—C11—H11	108.2	C22—C27—C26	121.64 (19)
C10—C11—H11	108.2	C22—C27—H27	119.2
C22—C11—H11	108.2	C26—C27—H27	119.2
C21—C12—C13	118.67 (19)	O1—C28—O2	107.74 (16)
C21—C12—C11	119.76 (19)	O1—C28—H28A	110.2
C13—C12—C11	121.44 (18)	O2—C28—H28A	110.2
C14—C13—C18	117.2 (2)	O1—C28—H28B	110.2
C14—C13—C12	123.07 (19)	O2—C28—H28B	110.2
C18—C13—C12	119.7 (2)	H28A—C28—H28B	108.5
C15—C14—C13	121.1 (2)		
			
C21—N1—C1—C10	12.1 (3)	C15—C16—C17—C18	0.5 (3)
C21—N1—C1—C2	−167.48 (19)	C16—C17—C18—C19	178.5 (2)
C10—C1—C2—C3	0.0 (3)	C16—C17—C18—C13	−0.9 (3)
N1—C1—C2—C3	179.57 (19)	C14—C13—C18—C17	1.0 (3)
C1—C2—C3—C4	−1.3 (3)	C12—C13—C18—C17	179.7 (2)
C2—C3—C4—C5	−177.5 (2)	C14—C13—C18—C19	−178.43 (19)
C2—C3—C4—C9	0.6 (3)	C12—C13—C18—C19	0.3 (3)
C3—C4—C5—C6	178.5 (2)	C17—C18—C19—C20	−179.0 (2)
C9—C4—C5—C6	0.4 (3)	C13—C18—C19—C20	0.4 (3)
C4—C5—C6—C7	−0.2 (4)	C18—C19—C20—C21	−0.3 (3)
C5—C6—C7—C8	−0.2 (4)	C13—C12—C21—N1	−179.68 (19)
C6—C7—C8—C9	0.4 (3)	C11—C12—C21—N1	−3.8 (3)
C5—C4—C9—C8	−0.2 (3)	C13—C12—C21—C20	1.1 (3)
C3—C4—C9—C8	−178.31 (19)	C11—C12—C21—C20	176.98 (18)
C5—C4—C9—C10	179.55 (18)	C1—N1—C21—C12	−14.5 (3)
C3—C4—C9—C10	1.4 (3)	C1—N1—C21—C20	164.76 (18)
C7—C8—C9—C4	−0.2 (3)	C19—C20—C21—C12	−0.5 (3)
C7—C8—C9—C10	−179.91 (19)	C19—C20—C21—N1	−179.7 (2)
N1—C1—C10—C9	−177.59 (17)	C12—C11—C22—C27	−125.2 (2)
C2—C1—C10—C9	1.9 (3)	C10—C11—C22—C27	111.1 (2)
N1—C1—C10—C11	8.4 (3)	C12—C11—C22—C23	58.9 (2)
C2—C1—C10—C11	−172.08 (19)	C10—C11—C22—C23	−64.9 (2)
C4—C9—C10—C1	−2.6 (3)	C27—C22—C23—C24	−1.8 (3)
C8—C9—C10—C1	177.10 (19)	C11—C22—C23—C24	174.26 (17)
C4—C9—C10—C11	171.23 (19)	C22—C23—C24—O1	179.2 (2)
C8—C9—C10—C11	−9.1 (3)	C22—C23—C24—C25	1.3 (3)
C1—C10—C11—C12	−23.9 (3)	C28—O1—C24—C23	172.2 (2)
C9—C10—C11—C12	162.39 (16)	C28—O1—C24—C25	−9.8 (2)
C1—C10—C11—C22	99.5 (2)	C23—C24—C25—C26	0.3 (3)
C9—C10—C11—C22	−74.3 (2)	O1—C24—C25—C26	−178.0 (2)
C10—C11—C12—C21	21.6 (2)	C23—C24—C25—O2	177.98 (18)
C22—C11—C12—C21	−100.5 (2)	O1—C24—C25—O2	−0.3 (2)
C10—C11—C12—C13	−162.66 (18)	C28—O2—C25—C26	−172.4 (2)
C22—C11—C12—C13	75.3 (2)	C28—O2—C25—C24	10.1 (2)
C21—C12—C13—C14	177.62 (19)	C24—C25—C26—C27	−1.3 (3)
C11—C12—C13—C14	1.8 (3)	O2—C25—C26—C27	−178.6 (2)
C21—C12—C13—C18	−1.0 (3)	C23—C22—C27—C26	0.7 (3)
C11—C12—C13—C18	−176.81 (17)	C11—C22—C27—C26	−175.17 (18)
C18—C13—C14—C15	−0.7 (3)	C25—C26—C27—C22	0.8 (3)
C12—C13—C14—C15	−179.4 (2)	C24—O1—C28—O2	15.9 (2)
C13—C14—C15—C16	0.3 (3)	C25—O2—C28—O1	−16.0 (2)
C14—C15—C16—C17	−0.2 (4)		

### Hydrogen-bond geometry (Å, °)

**Table:**

*D*—H···*A*	*D*—H	H···*A*	*D*···*A*	*D*—H···*A*
N1—H1···O2^i^	0.86	2.31	3.108 (2)	154
C5—H5···Cg^ii^	0.93	2.90	3.793 (2)	161

Symmetry codes: (i) *x*−1, *y*, *z*; (ii) *x*−1/2, −*y*+1/2, *z*−1/2.
